# Novel Method for Routine Ultrasound-Guided Serum Collection for Biomarker Analysis Around the Knee Joint

**DOI:** 10.12688/f1000research.159920.1

**Published:** 2025-01-13

**Authors:** Stefan Kluzek, Oliver O'Sullivan

**Affiliations:** 1 Centre for Sport, Exercise and Osteoarthritis Research, Versus Arthritis, Nottingham, England, UK; 2 Academic Unit of Injury, Recovery and Inflammation Sciences, School of Medicine, University of Nottingham, Nottingham, England, UK; 3 Academic Department of Military Rehabilitation, Defence Medical Rehabilitation Centre Stanford Hall, Loughborough, UK

**Keywords:** Biomarker, venous collection, lower-limb, joint, methodology, novel, osteoarthritis

## Abstract

**Background:**

Biomarkers are essential tools in modern medicine, allowing stratification and monitoring of clinical care and treatment response. While systemic blood biomarkers, typically collected from the antecubital vein (AV), are widely used, their sensitivity for joint-specific pathologies such as osteoarthritis (OA) may be limited due to systemic dilution. At present, no serum biomarker reliably reflects the microenvironment of an affected joint in clinical practice. Although synovial fluid (SF) assessment can provide insights into localised pathology and are clinically used for diagnosis in crystal arthropathies and joint infections, their collection is invasive, painful, and carries risks, including infection, making repeated sampling impractical, limiting utility for monitoring treatment responses. This study introduces a novel ultrasound-guided venous sampling technique targeting the saphenous vein (SV) proximal to the knee joint as a less invasive alternative to SF aspiration, hypothesising that it may better reflect the joint-specific microenvironment.

**Methods:**

A standardised gel model was used to train medically qualified researchers in ultrasound-guided venepuncture of vescles around 2-15 mm in diameter. Subsequently, 32 participants consented to blood sampling from the SV above the knee, with a proximally applied tourniquet to dilate vein diameter for easier venepuncture collection.

**Results:**

The technique achieved serum collection in over 80% of consented individuals with minimal adverse effects (including n=2 minor bruising and n=1 transient nerve irritation). Key procedural insights included optimal site selection, appropriate pressure application, and effective tourniquet use.

**Discussion:**

This method demonstrates feasibility, acceptability, and the potential for more localised sample collection, advancing biomarker research. Further validation, including paired SF comparisons, is required to confirm diagnostic utility and develop therapeutic strategies for joint-specific conditions.

## Introduction

Biomarkers, measurable indicators of biological states or conditions, have become integral to understanding, diagnosing, and treating diseases in contemporary medicine. The use of molecular biological markers (biomarkers) has allowed the prediction and stratification of clinical care, monitoring responsiveness to treatment and change on biological processes and has been a cornerstone of modern clinical medicine and pathophysiological research. ^1, 2^ These soluble markers, defined by the National Institute of Health *as a characteristic that is objectively measured and evaluated as an indicator of normal biological processes, pathogenic processes or pharmacological responses to a therapeutic intervention*, ^3^ are used as a proxy measure of a clinically relevant endpoint or pathological change. Those can be measured in biological fluids, including blood products (serum or plasma), synovial fluid (SF), cerebrospinal fluid (CSF), pleural effusion and urine. ^2^ The NIH classify them as:

- Type 0 (measure disease natural history),
- Type I (effect of an intervention, i.e., drug),
- Type II (surrogate endpoint markers).

Within the joint trauma and osteoarthritis (OA) field, there has been a particular focus on serum and plasma biomarkers to identify this common, disabling condition within the asymptomatic prodromal phase and as an outcome measure for pharmacological research studies. ^4– 8^ Research has focused on post-traumatic OA as a paradigm due to the clear initial event, younger age of the population and fewer co-morbidities. ^6, 9– 11^ Progress is slow within the field, partly due to lack of standardisation in collection and analysis methods, and differences across populations, biological fluids, and time from injury ^12, 13^; however, consensus is beginning to be reached. ^14, 15^ A further extended taxonomy of BIPEDS (Burden of Disease, Investigative, Prognostic, Efficacy of intervention, Diagnostic and Safety) has been created to classify OA-specific biomarkers, and tailor their use accordingly. ^11^

Biomarkers measured directly from the knee joint via SF provide valuable insights into the local microenvironment at the time of collection. ^16^ However, this procedure requires technical skill and is associated with several potential side effects, including pain and an increased risk of infection. Additionally, there is a risk of blood contamination during a traumatic tap, significant variability in biomarker concentrations due to hyaluronan concentration, and challenges in accurately measuring temporal changes in concentration. Therefore, as an alternative, systemic measurement of biomarkers from the blood is performed, which is technically easier, with fewer side-effects; however, paired serum and SF biomarkers correlate poorly, with no serum biomarker reliably reflecting the microenvironment of an affected joint. ^13^

The key challenge is the potential error in assuming that serum biomarkers collected from the upper arm reflect concentrations near an affected joint in the lower limb. The serum from the antecubital vein represents the fraction of the blood that, after leaving pulmonary circulation, is pumped from the left ventricle into the aorta and reaches the arm via the brachiocephalic or subclavian arteries before flowing through smaller arteries and capillaries and entering the venous system of the arm. However, this not the same in the lower limb, with the highly-vascularised synovial membrane providing effective plasma filtration and exchange with synovial tissue through an extensive network of capillaries, fed by fenestrated arterioles and venules in the synovium. These supply the capillary beds with blood flow, maintaining the pressure gradient and selective diffusion necessary for filtration, before the capillaries lead to venules which form the larger veins. ^17^ Transudate of plasma from synovial tissue blood vessels supplemented with high molecular weight saccharide-rich molecules, particularly hyaluronan. ^18^ Importantly, this ultrafiltrate of plasma, is actively reabsorbed to maintain homeostasis, ^17^ indicating that venous circulation from lower limb likely contains molecules that better reflect the knee, foot and ankle joint-specific processes than serum samples collected from upper limb.

Previous unpublished work has demonstrated that in those with Charcot-joint, levels of interleukin (IL)-6 were higher when measured in the venous system nearer the injured joint compared to further away. tThis might offer an opportunity to measure serum biomarker concentrations without systemic dilution which would better represent the local environment. To the author’s knowledge, there has been no previously described method to enable this. This manuscript aims to report the development of a novel technique to measure venous serum concentration near the knee joint to improve the ability to sample OA-related biomarkers. We also report on the identified challenges and complications, and provide insights from those trained in the new collection method.

## Methods

### Ethics and participants

The Faculty of Medicine and Health Sciences Research Ethics Committee, University of Nottingham (UoN FMHS 170-1122) granted a favourable ethical opinion for this study in April 2023. All researchers were trained in Good Clinical Practice and had appropriate phlebotomy and ultrasound skills. Sampling was performed at the Academic Unit of Injury, Recovery and Inflammation Sciences (IRIS), UoN, with chaperones in attendance during sampling (due to full exposure of the lower limb), between June and October 2023. Related data can be found here. ^19^

### Anatomical considerations

**Figure 1.**
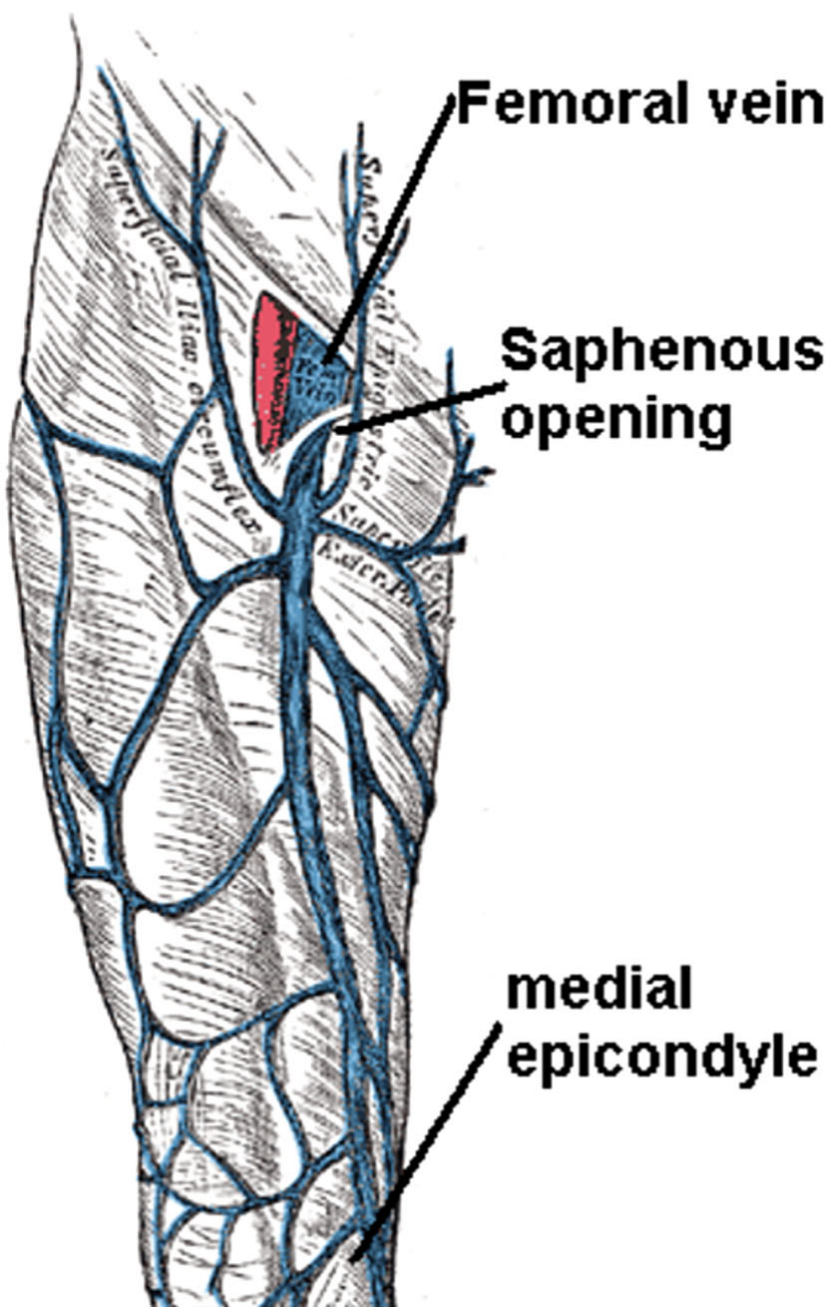
Venous circulation anatomy of the thigh. By Mikael Häggström, used with permission. ^30^

**Figure 2.**
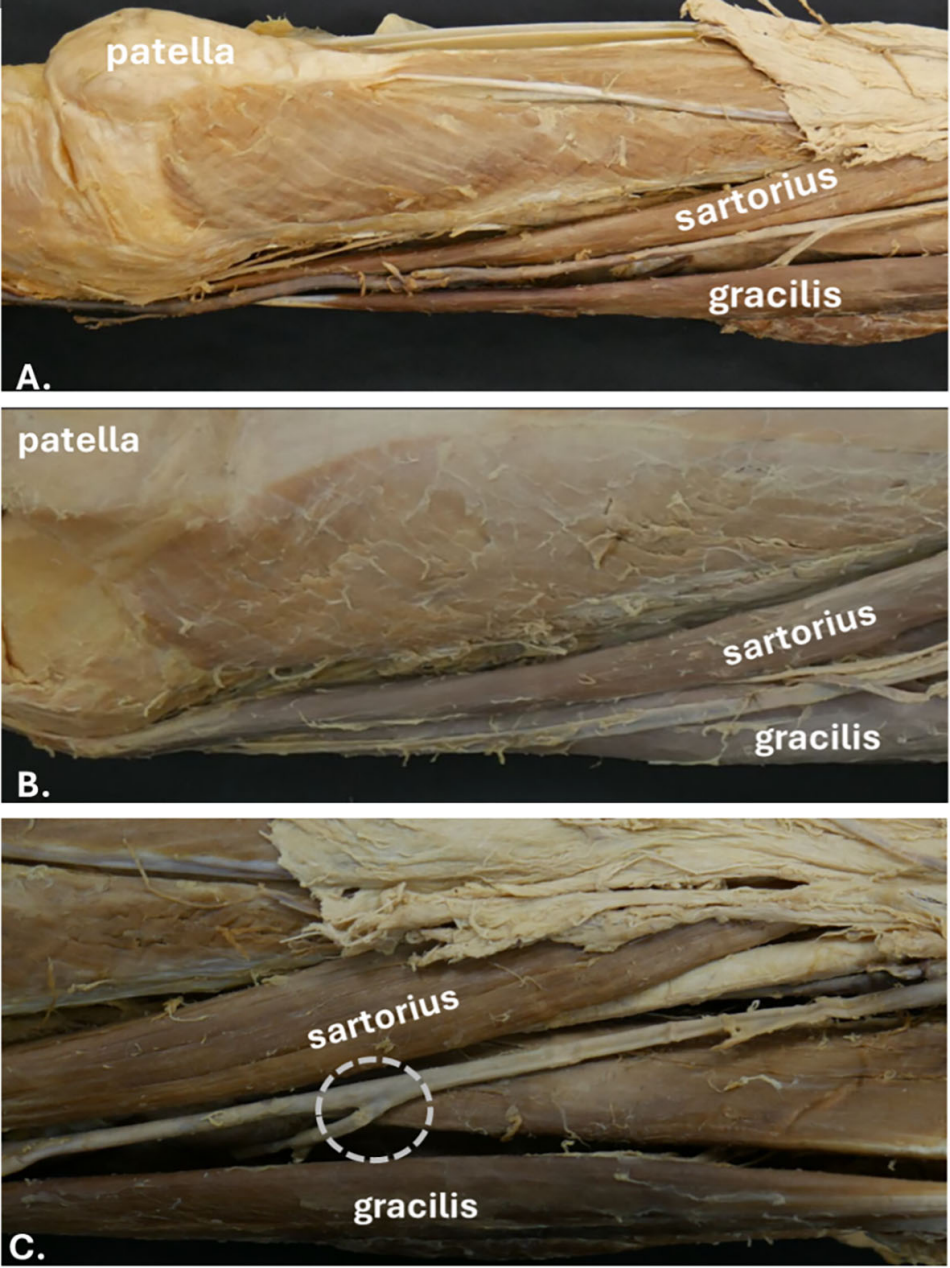
Position of the great saphenous vein (GSV), identified using the landmarks of the vastus medialis, sartorius, and gracilis muscles. A and B illustrate its consistency in two different donors, while C exemplifies its connection with the deep venous system.

**Figure 3.**
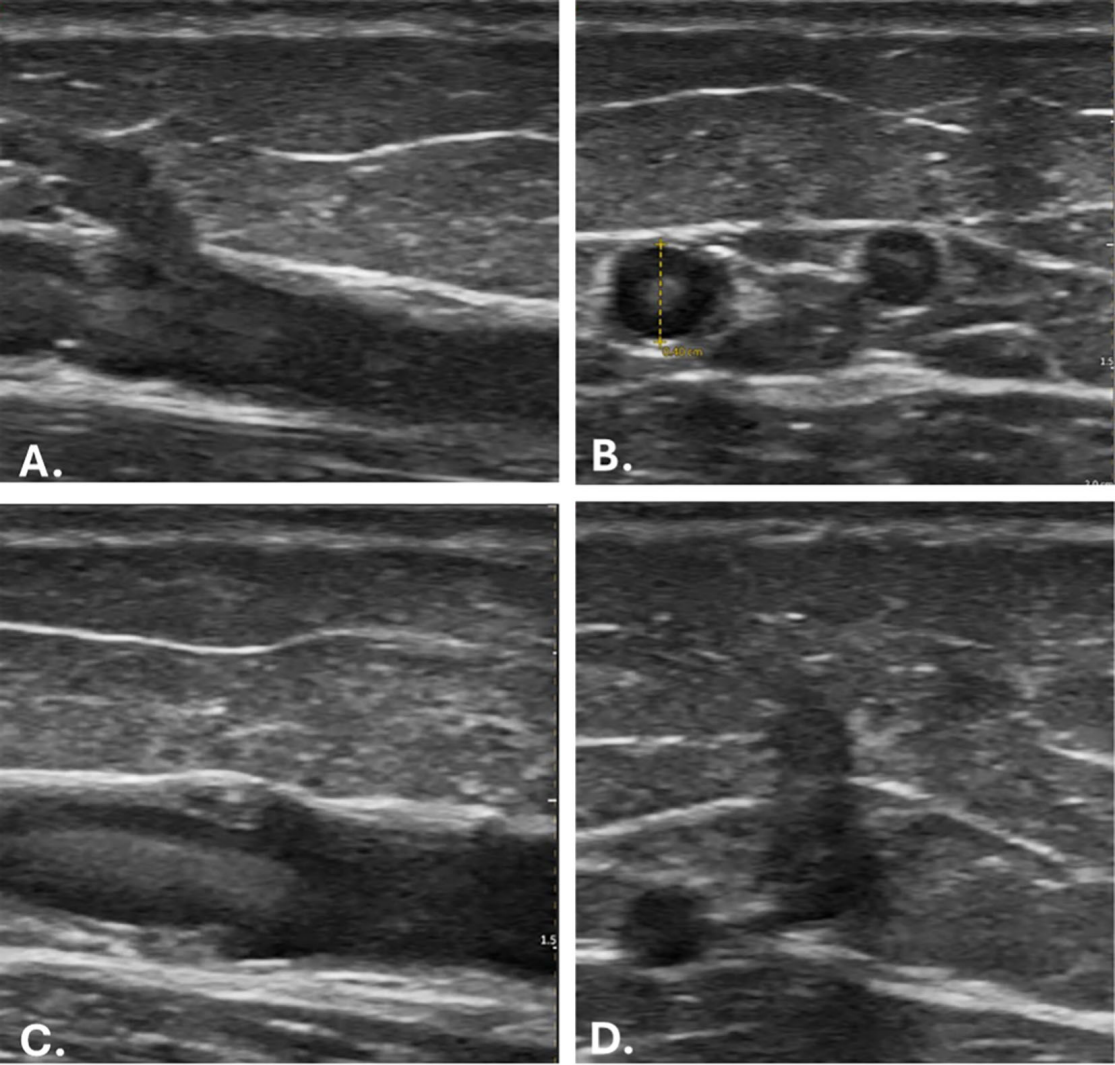
Ultrasound images of the great saphenous vein (GSV). A. Longitudinal view showing the superficial cutaneous branch. B. Cross-sectional view illustrating the smaller branch. C. Longitudinal view representing venous valves. D. Connection between the GSV and the deep circulation.

The greater saphenous vein (GSV) in the mid-thigh region (approximately 10–20 cm above the medial knee) was targeted for venous puncture ( Figure 1). It courses posterior to the medial femoral condyle, ascending medially towards its eventual junction with the femoral vein at the saphenofemoral junction (SFJ) in the groin ( Figure 2). The GSV runs along the medial aspect of the thigh, lying within the superficial fascia, and can be easily visualised with ultrasound ( Figure 3). It is important to note that just posterior to the medial condyle, anterior to the GSV, lies the saphenous nerve, which can be difficult to distinguish from connective tissue ( Figure 2). Above this region, the saphenous nerve diverges and follows a different course.

### Training

**Figure 4.**
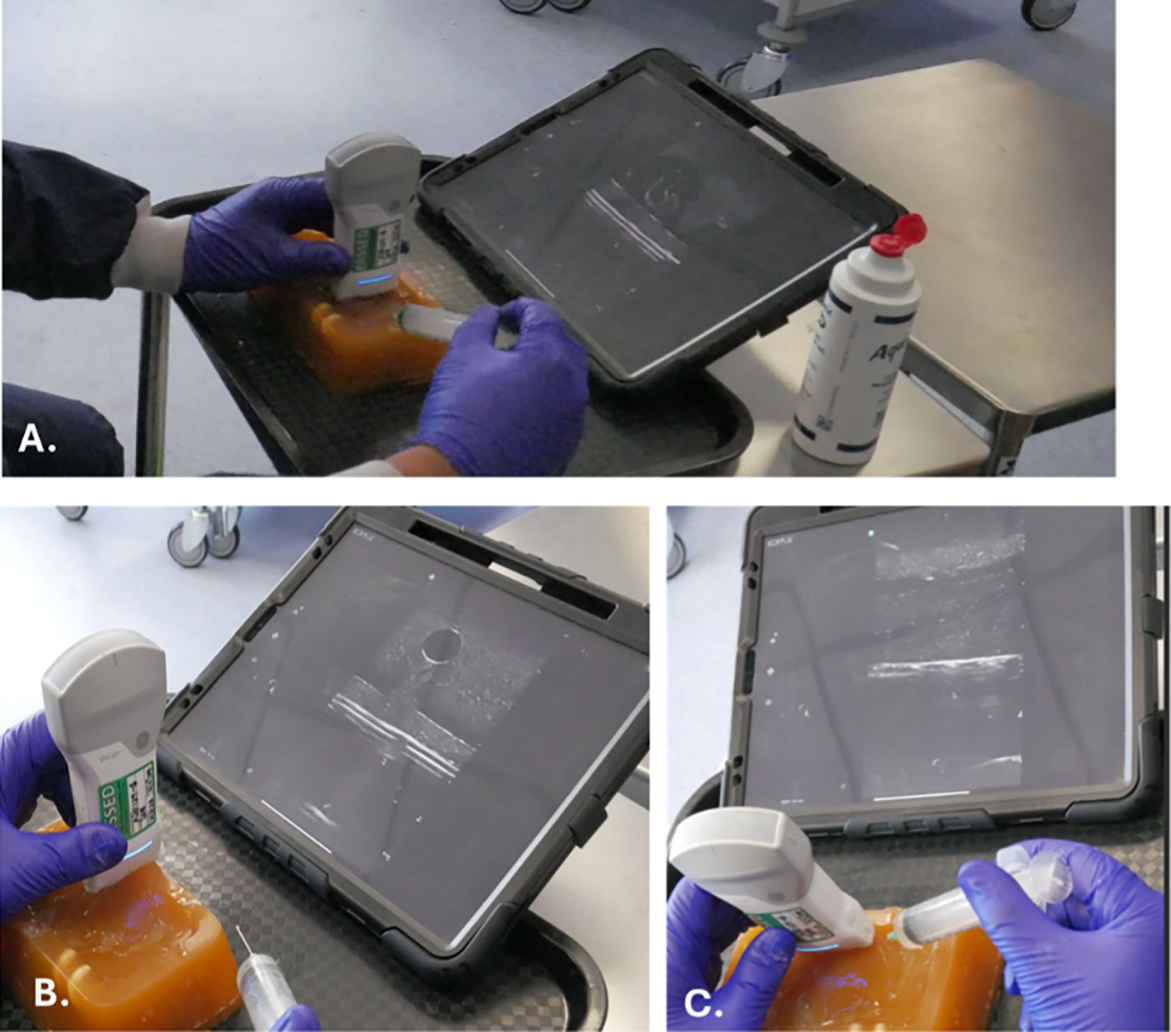
Set-up of the ultrasound gel training model for ultrasound-guided venepuncture, showing. A. General set-up, B. Out-of-plane venepuncture, and C. In-plane venepuncture.

**Figure 5.**
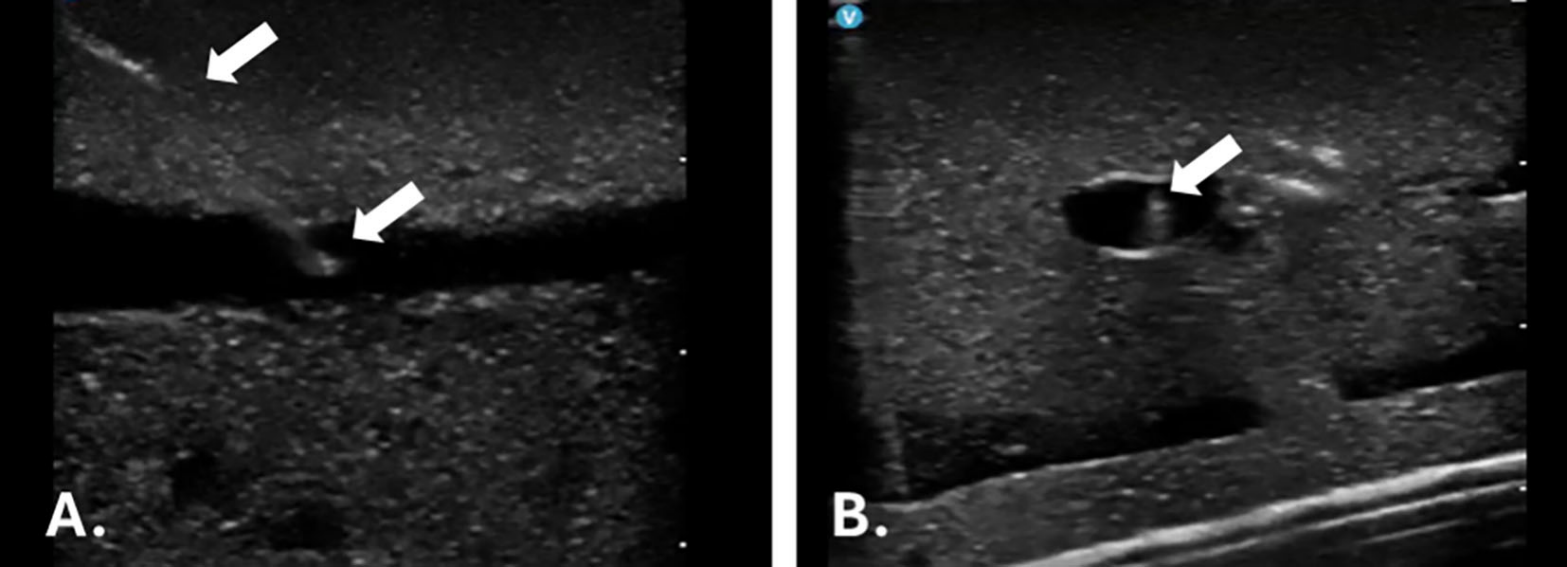
Ultrasound training in In-plane and Out-of-plane needle positioning and visualisation– images from the gel training model. A. Longitudinal view of the simulated vein in the gel model with in-plane needle placement (white arrows indicating the position of the needle). B. Cross-sectional view of the simulated vein in the gel model with out-of-plane needle placement (white arrows indicating the position of the needle).

Prior to performing this procedure on participants, the research team (experienced in phlebotomy) underwent training on a gel-based model to improve the ability to perform venepuncture under direct ultrasound visualisation ( Figure 4). ^20^ This was performed with a model made from clear ballistic gel utilising a previously described method. ^21^ The model contained a balloon partly filled with water, which aimed to replicate the images visible when performing ultrasound-assisted procedures ( Figure 5).

The training criteria were as follows:

1. Ensure trainees could operate the ultrasound device and optimise images using gain, depth, and focus.
2. Develop the ability to follow small cylindrical structures with diameters ranging from 2 to 15 mm.
3. Achieve proficiency in needle placement and control of needle visualisation during advancement of the needle, both in-plane and out-of-plane.
4. Successfully and repeatedly demonstrated the ability to aspirate fluid within the simulated vessels.

One researcher had limited prior experience using a handheld ultrasound device, highlighting the importance of adequate training to ensure competency. Two dedicated practice sessions using the gel-based model were sufficient to develop the necessary skills to perform ultrasound-guided venepuncture effectively. However, it is important to note that the level of training required may vary depending on the practitioner’s initial familiarity with ultrasound technology. For practitioners who are entirely ultrasound-naïve, additional practice sessions may be necessary to ensure a thorough understanding of the device and its application. Once the senior investigator was satisfied with the team’s proficiency, participant visits were scheduled.

The gel models were stored in a refrigerator between sessions, placed in a plastic container sealed with cling film to maintain hygiene and prevent contamination. Despite these precautions, one of the models developed visible mould on its top surface after three weeks of storage.

### Recruitment

Participants were recruited from the university, local sports clubs or networks. Study participation was voluntary, with written consent at least 24 hours after receiving the participant information leaflet.

### Positioning and marking

After informed consent and recruitment, participants were positioned on a pre-cleaned examination plinth reclined at 45 degrees, covered in a disposable blue roll. They were asked to wear shorts, with the lower limb fully exposed, and a foam roller placed under the knee to provide a mild degree of flexion and prevent unnecessary muscle contraction. The hip was mildly externally rotated and the knee slightly flexed.

When the participant was in position, comfort levels were verbally assessed as they needed to remain in position for upto 15 minutes. Once comfortable, the relevant anatomy was visualised. This was undertaken using a reusable rubber tourniquet applied over the upper third of the upper leg, with the lower limb venous vasculature identified using a hand-held ultrasound device (VScanAir™, General Electric, USA), which provided real-time images to a stand-alone portable device (iPad ^®^, Apple, USA). Real-time ultrasound was used to locate the proximal GSV and other relevant features (including depth from skin, vessel diameter, and presence of any anatomical variation). The ultrasound settings, including gain, depth, and focus, were optimised to ensure clear imaging of the vein, which allows precise targeting during the procedure. The overlying skin was marked using a washable marker pen, and the tourniquet was loosened while the sampling pack was assembled ( Box 1).

Box 1. **Equipment required for novel lower-limb venepuncture.**

• Blue (23G) and green (21G) needles, depending on the vein’s size and accessibility
• 2 mL, 5 mL, and 10 mL capacity syringes, depending on required sample volume
• Vacuum-tube collection system was also used, when appropriate
• Hospital/department-approved needle/sharps container and clinical waste disposal
• A sterile pack containing drapes, ultrasound gel, a probe cover, and bands
• Long green re-usable rubber venous tourniquet
• 70% isopropyl alcohol wipes
• Antiseptic 2% Chlorhexidine Gluconate/70% Isopropyl Alcohol formulation
• Sterile gloves
• Suggested (based on participants’ comments): a warm pack and/or topical anaesthetic gel to minimize discomfort during the procedure

### Procedure

The procedure began with thorough handwashing using World Health Organization (WHO) techniques before donning double sterile gloves. ^22^ To maintain a sterile field and prevent cross-contamination, sterile drapes, sterile ultrasound gel, and a sterile probe cover were used throughout the procedure. The injection site was cleansed with 70% isopropyl alcohol wipes, followed by the application of an antiseptic, 2% chlorhexidine gluconate and 70% isopropyl alcohol containing, cleaning product (Chloraprep™, Becton, Dickinson and Company (BD), UK).

### Venepuncture

The needle was placed under direct ultrasound guidance, using either an in-plane or out-of-plane approach depending on the vein’s orientation ( Figure 5). The needle was advanced carefully under visualisation to minimise the risk of complications. Three attempts were undertaken during sampling - if the third attempt was unsuccessful, the researcher terminated the procedure. Once the sample had been collected, serum was transferred to analysis tubes, the participant checked for any post-procedure side effects, with equipment was tidied away and cleaned using alcohol wipes for future use.

After the procedure, pressure was applied to the puncture site to prevent bleeding, and the area was dressed appropriately. All used equipment was disposed of, following clinical waste protocols.

## Results

During the pilot development of this procedure, 32 participants underwent sampling by two researchers (OOS and SK), with 81.2% of procedures successfully collecting serum. The proportion of sampling procedures requiring multiple attempts remained similar throughout (first 16 participants, 50%, second 16 participants, 50%). From the successful procedures (n=26), fifteen were collected with one attempt (58%), eight after two attempts (31%), and five required three attempts (19%) ( Table 1). Two participants underwent three unsuccessful attempts without collection (6%). Four participants (12%) asked to terminate during the sampling procedure due to pain before successful completion (first attempt n=2, second attempt n=1, third attempt n=1) ( Table 1). Aside from these, no major side effects or adverse events were encountered during or after the procedure.

**Table 1.** Number of attempts during successful procedures.

Procedure attempt	Number (n=, %)
1	15 (58%)
2	8 (31%)
3	5 (19%)

### Comments from the researchers and participants

Needle Positioning: “When inserting the needle toward the knee, it is recommended to rest the wrist on the leg for better control. This technique is likely no to create a pressure decrease observed when needle is inserted away from the knee, which facilitates fluid collection.”

Success Rate: “The procedure tends to be more successful when the needle is positioned higher and above the valve connecting saphenous vein with deep circulation above the knee.”

Saphenous Vein Caution: “The saphenous vein is located close to the nerve just above the knee, but it then diverges. Careful needle placement is necessary to avoid nerve irritation”.

Application of topical anaesthetic: “Applying a heat pack and/or topical lidocaine gel before the procedure could be very helpful in reducing stress and discomfort.”

## Discussion

This paper examines the feasibility and acceptability of a novel lower-limb venous sampling technique, which may provide valuable insights into the microenvironment of lower-leg metabolism, particularly in affected joints. As the first description of this technique, the discussion focuses on the development and refinement of the procedure, researcher training, and potential areas for future improvement and relevance.

Given the technical requirements of this procedure, appropriate training is essential. All the research team were experienced and proficient in phlebotomy, though one researcher only had limited experience with handheld ultrasound device. Two practice sessions using the gel model was sufficient to develop these skills. However, additional training may be required for future ultrasound-naïve practitioners.

During the development of this technique, the equipment used was refined and optimised. Different tourniquet models were trialed, including disposable thin rubber, reusable fabric and rubber tourniquets. Venepuncture in the lower limb presents unique challenges compared to the antecubital fossa, such as accommodating the limb’s larger diameter and applying sufficient pressure to occlude deeper vessels. The disposable thin rubber tourniquet was variously too short and snapped under sustained pressure, and the fabric reusable tourniquet was impracticable short. The re-usable rubber tourniquet (daisygrip™, Tristel, UK) maintained the required increased pressure and had the length required for all participants’ thigh diameters. It was cleaned between each use, essential for infection prevention. A further refinement might be using a blood flow restriction cuff, given its increased use in rehabilitation pathways. ^23^

Other equipment utilised and refined throughout this development was the needle and syringe combination. Initially, a butterfly system directly attached to the vacuum specimen tube (Vacutainer ^®^, BD, UK) was trialled; however, often, the needle was not long enough to reach the subcutaneous or deeper vessels, and the pressure by the vacuum-tubes itself was too high for the small, fragile veins, causing vein collapse and reduced blood flow. As a result, the research team adopted the needle and syringe combination, which provided several advantages. The adjustable pressure allowed for gentler handling of fragile veins, and the longer needle offered better access to deeper vessels. Additionally, the team experienced improved dexterity and control with this system, particularly beneficial when sampling from deep or small-calibre veins. This refined approach enhanced the precision and success rate of blood collection in challenging cases.

Despite its experimental nature, it was notable how acceptable and tolerable participants found this procedure. All participants had the technique explained in the participant information leaflet and initial brief, and once informed, no participants declined, suggesting this procedure was deemed acceptable. During this pilot, there were 59 attempts across all participants, with four of them causing pain sufficient to request termination (7%), two cases of post-procedure bruising and no other side or post-venepuncture effects, demonstrating patient tolerability. In the literature, adverse reactions have been recorded in up to 49% of venepuncture procedures ^24^ with the most common side effect, pain, improved through the use of topical analgesia. ^25^ However, the pain of this procedure is likely due to the regional anatomy – specifically, the proximity of the saphenous nerve to the GSV, ^26^ with a more proximal approach likely to reduce the risk of severe pain.

The success rate of the procedure was high, with only two episodes of unsuccessful blood sample collection despite three attempts (6%); however, half the participants underwent multiple attempts. There are two key reasons underpinning this, participant factors and operator factors. When the most suitable blood vessel was detected on ultrasound, those located deeper from the skin and those with the smallest calibre were typically harder to cannulate. During the early stages, vessels under 3 mm had a lower success rate, however, by the end, those above 2.5 mm were considered large enough. Whilst it is interesting to note that first-attempt operator success remained at approximately 50% through the study, this would likely increase with more operator experience and competence, as potentially demonstrated by the anecdotal experiences related to vessel diameter. A further area that might improve rates for those with deeper vessels or high volumes of subcutaneous adipose tissue is the use of a longer needle length, which was not available to the research team during this pilot study.

This novel technique for venepuncture nearer to the knee joint offers an opportunity to improve OA biomarker research across all elements of the BIPEDS taxonomy, especially for those with a lower-limb injury. ^9, 11^ It is likely that any biomarkers formed and/or released by the synovium or other components of the knee or ankle joint undergo dilutional effects in the systemic circulation, thus reducing their sensitivity. ^27, 28^ Differences were seen in the concentrations of biomarkers measured in samples from the saphenous vein and antecubital vein, with detailed findings described in a separate publication. ^29^ If thresholds of biomarker concentration are used to determine ‘normal vs abnormal’ and identify or define a diagnosis, then reduced concentration and sensitivity will cause false negative results. Furthermore, there is a poor correlation between paired serum and synovial fluid biomarkers, which might result from dilution or perhaps degradation of the serum biomarker. ^13^ A further study undertaking paired local-serum and SF samples is required to assess if this novel technique improves correlation. Subsequent work, such as radioisotope tracing (e.g., technetium-99m) or imaging techniques like gamma scintigraphy, also offers the potential to clarify venous return dynamics and validate the GSV.

In conclusion, this manuscript reports the development of a novel technique to perform lower-limb venepuncture. This acceptable and well-tolerated technique, with a small learning curve and easily available low-cost equipment, might offer a way to provide insight into the joint microenvironment without the risks associated with SF sampling and, therefore, improve the use of serum biomarkers for OA.

## Ethics and consent

Ethical approval was granted by the Faculty of Medicine and Health Sciences Research Ethics Committee, University of Nottingham (UoN FMHS 170-1122) in April 2023. Participants provided informed, written consent. Consent was granted by the researchers featured in the photographs within the manuscript. This study was conducted in line with the Declaration of Helsinki.

## Author contributions

SK and OOS were equally involved in conceptualization, data acquisition, curation and analysis, and writing (drafting and editing). In addition, SK led on funding acquisition and provided supervision.

## Data Availability

Data and software codes are freely-available and accessible. They are posted Open Source via the GitHub platform, with a digital objector identifier created using Zenodo. Data is under a CC BY 4.0 license. STATA 18.5 (STATACorp, LLP), was used for analysis, free software analysis alternatives include R ( https://www.r-project.org/). Repository: GitHub. “Novel Method for Routine Ultrasound-Guided Serum Collection for Biomarker Analysis Around the Knee Joint” Link: https://github.com/stefankluzek/SOREstudy DOI: https://doi.org/10.5281/zenodo.14454447. ^19^ This link contains the following underlying data:
• README.md – Authors, contents and key variables • SORE analysis 21102024.do – code used to analyse date (for STATA, version 18.5) • SORE lab data.xlsx – raw results of serum biomarker assays • SORE participant information leaflet template.docx • SORE consent form 2021 v1.1.docx • SORE pre-screening questionnaire.docx • STROBE checklist.docx Data are available under the terms of the Creative Commons Attribution 4.0 International license (CC-BY 4.0).
