# Supplementary material for: Novel Method for Routine Ultrasound-Guided Serum Collection for Biomarker Analysis Around the Knee Joint
Source: F1000Res. 2025 Jan 13;14:68. [Version 1] doi: 10.12688/f1000research.159920.1 (PMC12398681; doi:10.12688/f1000research.159920.1)
Supplement: Peer review history

# Reviewer report: Reviewer response for version 1

**DOI:** 10.5256/f1000research.175713.r399999

**Published:** 2025-08-30

**Authors:** Anne Bay-Jensen

**Affiliations:** 1 Nordic Bioscience, Herlev, Denmark

I have reviewed the manuscript entitled: "Novel Method for Routine Ultrasound-Guided Serum Collection for Biomarker Analysis Around the Knee Joint". The topic is valid and important. The main miss in the paper is evidence that taking blood from SV provide more accurate measurement than blood for AV.

 One could argue that the high rate of failed attempts (table 1) is not compatible with taking AV blood. Are there any official numbers to AV failed attempts?

 In the introduction, the authors refer to the NIH biomarker criteria, but I would suggest to use the terminology of the NIH-FDA BEST resource, as this more precisely describe the path of various type of biomarkers ( https://www.ncbi.nlm.nih.gov/books/NBK326791).

 In the conclusion the authors suggest to compare SV blood levels with SF levels of molecular biomarkers, but it will be equally important to look at levels from SV and AV, to understand the win by changing to the SV methodology from the more standard AV blood sampling.

Is the rationale for developing the new method (or application) clearly explained?

Yes

Is the description of the method technically sound?

Yes

Are the conclusions about the method and its performance adequately supported by the findings presented in the article?

Yes

If any results are presented, are all the source data underlying the results available to ensure full reproducibility?

Yes

Are sufficient details provided to allow replication of the method development and its use by others?

Yes

Reviewer Expertise:

Soluble biomarker research

I confirm that I have read this submission and believe that I have an appropriate level of expertise to confirm that it is of an acceptable scientific standard.

# Reviewer report: Reviewer response for version 1

**DOI:** 10.5256/f1000research.175713.r372319

**Published:** 2025-04-01

**Authors:** Fiona Saunders

**Affiliations:** 1 University of Aberdeen, Aberdeen, Scotland, UK

This is an interesting paper which investigates the feasibility of blood sampling from the saphenous vein as a means of collecting serum for analysis of biomarkers for osteoarthritis in the knee joint. The authors have clearly explained the rationale for the development of this method, outlining the poor correlation that standard methods have with biomarker concentrations. Technically, the methodology seems sound, with acknowledgement of the training and experience that would be required for this methodology to be accepted into research practice. There is sufficient detail that the methodology would be able to be reproduced by others. The authors do not include any details of the ages and sex of the volunteers who participated in the study. These details are quite important as this could reflect the ease with which sampling was performed. This is likely to be easier in young, fit healthy participants, however the likelihood is that this method will be used to assess biomarkers in older, more medically frail adults, perhaps the authors could comment on this as a limitation of the methodology. The authors could also provide more information on adjustments that may need to be made to this protocol for it to be applied to older participants. 

 I have a few more specific comments on the manuscript.

 1.  In the abstract methods vessels is spelt incorrectly. 2. In the introduction, the sentence beginning "Transudate of plasma..." does not make any sense, it looks like a verb is missing.

 3. There is a typo in the next paragraph tThis.

 4. I think it should be made clear that the images from figure 2 are from prosections.

 5. How was the proficiency in the technique assessed and was there any input from experienced phlebotomists and clinicians/sonographers?

 6. Can the authors comment on whether volume of blood drawn made a difference to the pain reported by the participants or was the pain just due to the needle being inserted? 

 7. Can the authors comment on the suitability of the gel model for training and would there be any benefit using cadaveric/prosection material in addition?

Is the rationale for developing the new method (or application) clearly explained?

Yes

Is the description of the method technically sound?

Yes

Are the conclusions about the method and its performance adequately supported by the findings presented in the article?

Yes

If any results are presented, are all the source data underlying the results available to ensure full reproducibility?

Partly

Are sufficient details provided to allow replication of the method development and its use by others?

Yes

Reviewer Expertise:

Osteoarthritis

I confirm that I have read this submission and believe that I have an appropriate level of expertise to confirm that it is of an acceptable scientific standard.
